# CD147 promotes Src-dependent activation of Rac1 signaling through STAT3/DOCK8 during the motility of hepatocellular carcinoma cells

**DOI:** 10.18632/oncotarget.2801

**Published:** 2014-11-16

**Authors:** Shi-Jie Wang, Hong-Yong Cui, Yan-Mei Liu, Pu Zhao, Yang Zhang, Zhi-Guang Fu, Zhi-Nan Chen, Jian-Li Jiang

**Affiliations:** ^1^ Cell Engineering Research Center & Department of Cell Biology, State Key Laboratory of Cancer Biology, National Key Discipline of Cell Biology, Fourth Military Medical University, Xi'an, China; ^2^ College of Life and Health Sciences, Northeastern University, Shenyang, China

**Keywords:** CD147, Src, Rac1, DOCK8, STAT3

## Abstract

Metastasis is considered a dynamic process in tumor development that is related to abnormal migration and invasion. Tumor cells can move as individual cells in two interconvertible modes: mesenchymal-type and amoeboid. Previously, we reported that the interaction between CD147 and Annexin II can inhibit the amoeboid movement in hepatocellular carcinoma (HCC) cells. However, the mechanism of CD147 involved in mesenchymal movement is still unclear. Notably, our results show overexpression of CD147 led to mesenchymal-type movement in HCC cells. Evidence indicated that the mesenchymal-type cell movement induced by CD147 was Src dependent, as observed by confocal microscopy and Rac1 activity assay. The phosphorylation of Src (pY416-Src) can be up-regulated by CD147, and this regulation is mediated by focal adhesion kinase (FAK). Next, we identified DOCK8 as a GEF for Rac1, a key molecule driving mesenchymal-type movement. We also found that Src promotes STAT3 phosphorylation and STAT3 facilitates DOCK8 transcription, thus enhancing DOCK8 expression and Rac1 activation. This study provides a novel mechanism of CD147 regulating mesenchymal-type movement in HCC cells.

## INTRODUCTION

The metastatic dissemination of tumors is the main cause of death for most cancer patients [1, 2]. Tumor metastasis is a highly complex and multistep process that requires a tumor cell to modulate its ability to adhere, invade into the surrounding extracellular matrix, migrate, and proliferate at a secondary site [3]. Migratory cancer cells undergo dramatic molecular and cellular changes by remodeling their cell-cell, cell-matrix adhesion, actin cytoskeleton, and molecular processes involved in various signaling networks [2, 4, 5]. Two different, interconvertible modes of cell movement led by two unique signaling pathways have been demonstrated in individual tumor cells [6, 7]. Mesenchymal-type movement is characterized by an elongated cellular morphology that results from Rac-dependent actin assembly at the leading edge and requires extracellular proteolysis for Rac-dependent actin protrusions to be pushed through channels in the extracellular matrix. In contrast, amoeboid movement is characterized by a rounded, blebbing morphology that is independent of extracellular proteases [8-10]. These two signaling pathways coordinate to regulate the cytoskeletal rearrangement that plays a critical role in the movement mode transition.

CD147, which belongs to the immunoglobulin superfamily, is a glycosylated transmembrane protein that is highly expressed in various cancers [11, 12]. Overexpression of CD147 can enhance the metastatic potential of cancer cells due to increased adhesion, migration, invasion, and matrix metalloproteinases (MMPs) secretion [13-15]. Our previous study indicated that CD147 inhibits Rho signaling pathways and amoeboid movement by inhibiting Annexin II phosphorylation [16].

The non-receptor tyrosine kinase Src is overexpressed and activated in many human malignancies [17, 18]. This kinase associates with many crucial aspects of tumor development and regulates a number of signaling pathways that are involved in survival, adhesion, invasion, migration, and proliferation [19-21]. In addition, deregulation of Src leads to constitutive kinase activation and cellular transformation. Decades of research have revealed the crucial role of Src in regulating small GTPases, and one of the major functions of Src is to modulate the actin cytoskeleton that controls cell migration [22]. However, it remains unknown whether Src is involved in the interconversion between the two modes of individual cell movement and the underlying mechanisms are far from clear.

In the present study, our results demonstrated that CD147 modulates Src activity through FAK when regulating cytoskeletal rearrangement and cell movement. We identified DOCK8 as a GEF for Rac1, which drives mesenchymal-type cell movement. Furthermore, Src promotes STAT3 phosphorylation and STAT3 facilitates DOCK8 transcription, thus enhancing DOCK8 expression and Rac1 activation.

## RESULTS

### CD147 promotes mesenchymal-type cell movement

**Fig.1:**
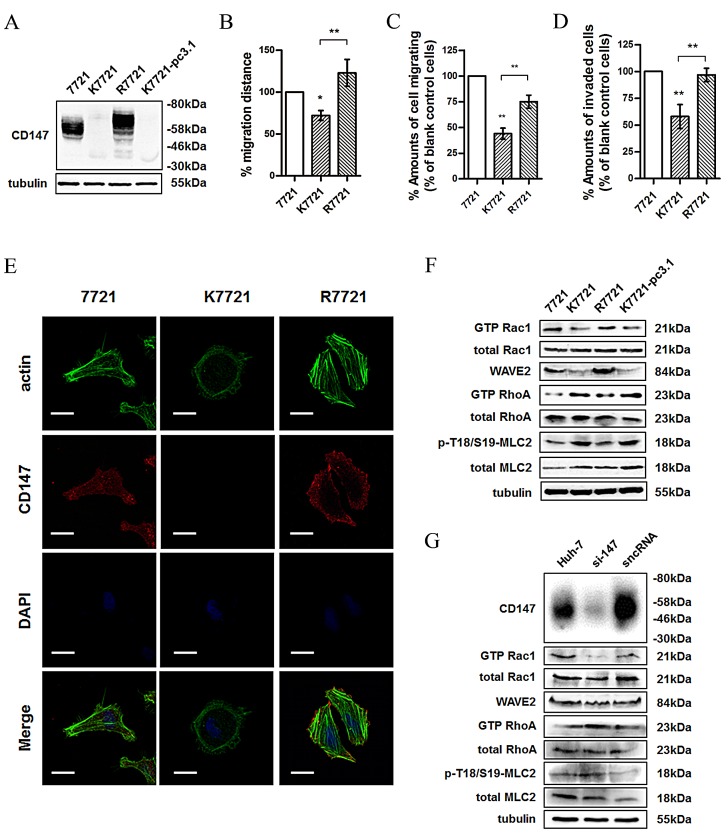
CD147 regulates cell morphology and motility via coordinating Rac and Rho signalings (A) CD147 was examined in total lysates of 7721, K7721, R7721 and K7721-pcDNA3.1 cells (K7721 cells transfected with pcDNA3.1 vector as a mock control for R7721) using western blotting. (B) Wound healing assay of the 7721, K7721 and R7721 cell lines. (C) Migration assay of the 7721, K7721 and R7721 cell lines. (D) *In vitro* invasion assay of the 7721, K7721 and R7721 cell lines. (E) Confocal microscopy images of 7721, K7721 and R7721 cells. Red: CD147; Green: actin; Blue: DAPI. Scale bar = 20μm. (F) Rac1 activity, WAVE2 expression, and RhoA and MLC2 activities were examined in total lysates of 7721, K7721, R7721 and K7721-pcDNA3.1 cells using western blotting. (G) Src and Rac1 activities, WAVE2 expression, and RhoA and MLC2 activities were examined in total lysates of Huh-7 cells and Huh-7 cells transfected with si-147 or sncRNA using western blotting. The bars represent each sample performed in triplicate, and the error bars indicate ± SD. ** P < 0.01, * P < 0.05, by one-way ANOVA (B-D).

To better understand the role of CD147 in regulating cell morphology and motility, we established a CD147-knockout 7721 cell line (K7721) using the zinc finger nuclease (ZFN)-targeting approach [23]. We first evaluated the expression of CD147 in 7721, K7721 and R7721 cells (Fig. 1A). As shown in Fig. 1B and 1C, cell migration was significantly decreased when CD147 was knocked out, whereas the ability was restored when the expression of CD147 was recovered. We also assessed the invasion ability of the cells, and the results shown in Fig. 1D were consistent with those of the migration assay. Morphological changes were also observed as shown in Fig. 1E; depletion of CD147 produced a more rounded morphology with prominent cortical F-actin in K7721 cells, while R7721 cells displayed a more elongated morphology with bundled F-actin. These results indicated that these changes in cell morphology and motility were due to the loss of CD147.

Mesenchymal-type and amoeboid movements are recognized as interconvertible modes when adapting to different microenvironments and are regulated by the Rac and Rho signaling pathways, respectively. We previously reported that CD147 promotes the cytoskeletal rearrangement and cell motility in HCC cells. Here, we examined the molecules related with mesenchymal-type and amoeboid movements and found that Rac1-GTP and WAVE2 expression were reduced, while RhoA-GTP and MLC2 phosphorylation were increased, following the depletion of CD147 (Fig. 1F). These results proved that CD147 is involved in the interconvertible cell movement. Similar results were obtained when CD147 was silenced in Huh-7 cells (Fig. 1G).

### Inhibition of Src leads to cell morphology and motility changes in HCC cells

**Fig.2:**
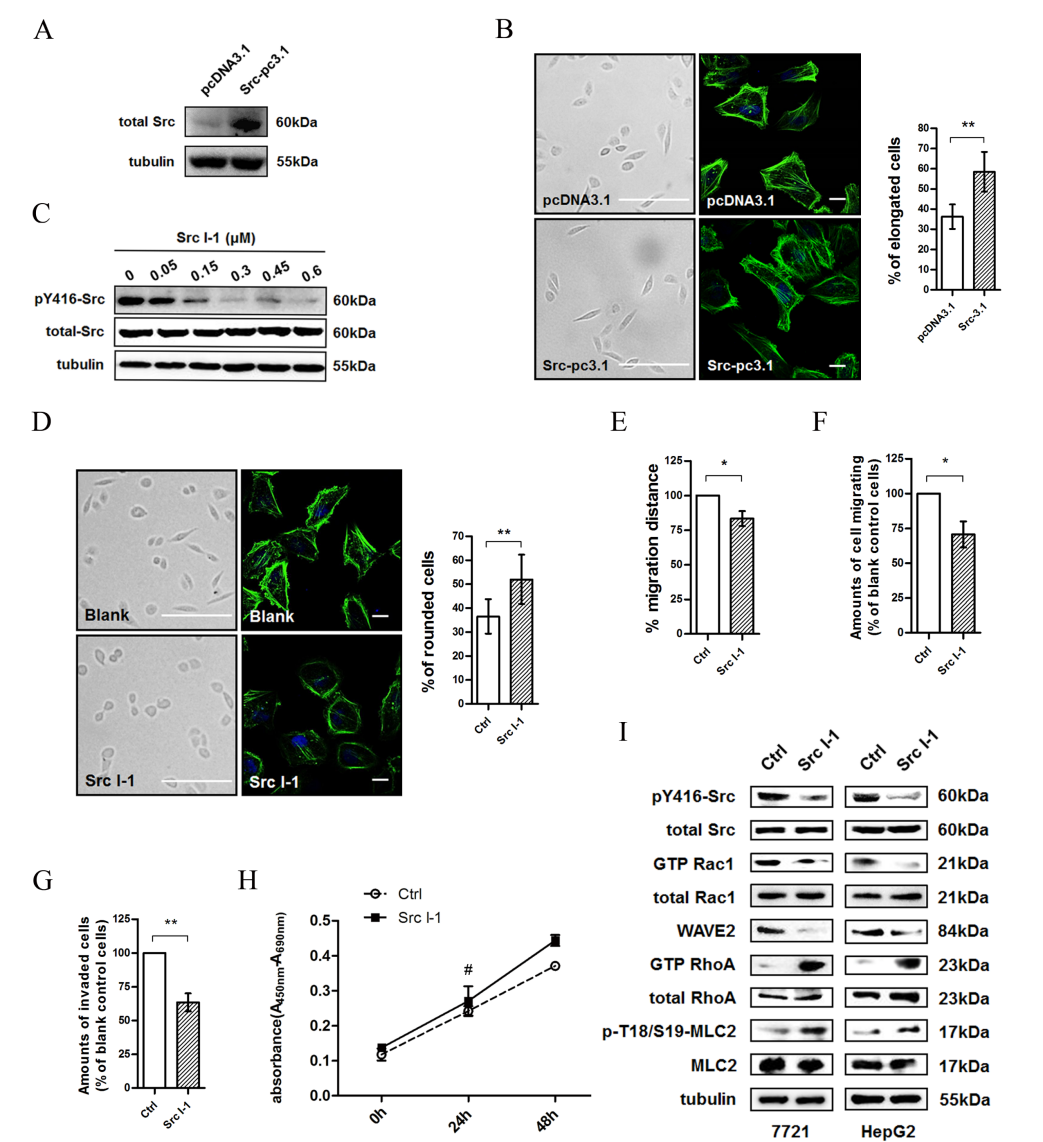
Src activity alteration leads to morphological and migratory activity changes in HCC cells (A) Src activity level (pY416-Src/total Src) was assessed using western blotting in pcDNA3.1 or Src-pc3.1 transfected 7721 cells. (B) Images (Scale bar = 500μm) and confocal microscopy images (Scale bar = 20μm) demonstrating the effect of Src overexpression on morphological changes in 7721 cells. Green: actin; Blue: DAPI. Left panel: representative image. Right panel: quantification. (C) Src activity was assessed using western blotting after treatment of 7721 cells with Src kinase inhibitor (Src I-1). (D) Images (Scale bar = 500μm) and confocal microscopy images (Scale bar = 20μm) demonstrating the effect of Src I-1 treatment on morphological changes in 7721 cells. Green: actin; Blue: DAPI. Left panel: representative image. Right panel: quantification. (E) Effects of Src I-1 treatment on cell motility of 7721 cells. (F) Effects of Src I-1 treatment on cell migration of 7721 cells. (G) Effects of Src I-1 treatment on cell invasion of 7721 cells. (H) Effects of Src I-1 treatment on cell proliferation of 7721 cells. (I) Src and Rac1 activities, WAVE2 expression, and RhoA and MLC2 activities were examined in 7721 and HepG2 cells treated with 300 nM of Src I-1. The bars represent each sample performed in triplicate, and the error bars indicate ± SD. ** P < 0.01, * P < 0.05, # P > 0.05, by t-test (B, D-G) and one-way ANOVA (H).

We first evaluated the effects of Src overexpression on cell morphology. A confocal fluorescence microscopy assay showed that overexpression of Src (Fig. 2A) resulted in a more elongated morphology with prominent cortical F-actin expression (Fig. 2B), which is consistent with mesenchymal-type movement. Then we investigated whether Src plays a dominant role in the changes of cell morphology. Results showed that inhibition of Src activity by Src I-1 (Fig. 2C), one of the gold standards for Src kinase inhibition [24], resulted in a more rounded morphology of 7721 cells (Fig. 2D), which is consistent with amoeboid movement. Wound healing and migration assays revealed that the migration ability of 7721 cells treated with Src I-1 was decreased compared to the solvent control group (Fig. 2E, 2F). In addition, the invasion ability was also significantly down-regulated after Src inhibition (Fig. 2G) without obvious alteration in cell proliferation (Fig. 2H). Interestingly, these phenomena were similar to the phenotype observed after inhibiting CD147 expression and Rac1 signaling pathway as shown in Fig. 1. We next evaluated whether Src is involved in coordinating the Rac/Rho signaling pathway in HCC cells. As shown in Fig. 2I, Src I-1 treatment decreased Rac1 activity (GTP Rac1/total Rac1) and WAVE2 expression in 7721 and HepG2 cells, which also substantially increased RhoA activity (GTP RhoA/total RhoA) and MLC2 activity (p-MLC2/MLC2). These results suggested that Src promotes the Rac1 signaling pathway but inhibits the RhoA signaling pathway in cytoskeletal rearrangement and cell movement in HCC cells, a role similar to that of CD147.

### Src is required for CD147-regulated cell movement in HCC cells

**Fig.3:**
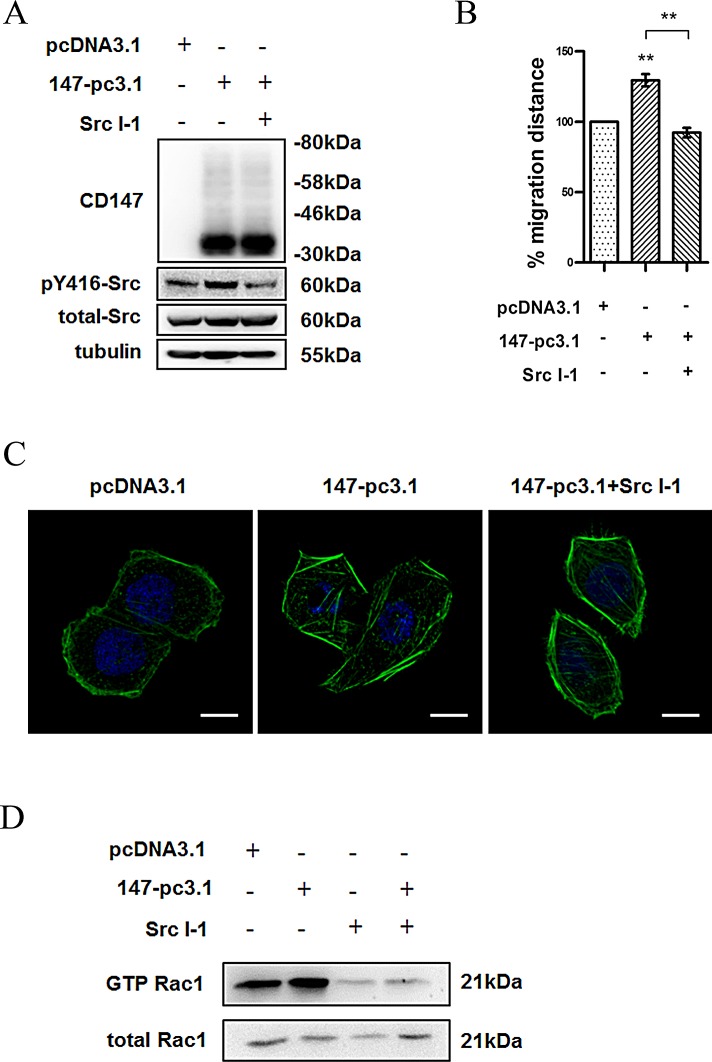
Src is required for CD147 regulated cell movement in HCC cells (A) Src activity in CD147-pcDNA3.1-expressing K7721 cells treated with Src I-1. (B) Effects of CD147 overexpression and/or Src I-1 treatment on cell motility in 7721 cells. (C) Confocal microscopy images (Scale bar = 10μm) of CD147 overexpression and/or Src I-1 treatment on cytoskeleton rearrangement in 7721 cells. (D) Rac1 activity in CD147-pcDNA3.1-transfected 7721 cells. The bars represent each sample performed in triplicate, and the error bars indicate ± SD. ** P < 0.01, by one-way ANOVA (B).

To verify the functional association between CD147 and Src in cell movement, we transfected K7721 cells with CD147-pcDNA3.1, and the transfected cells were treated with or without the Src inhibitor Src I-1 (Fig. 3A). As expected, the Src inhibitor blocked the CD147-enhanced cell motility (Fig. 3B) and cytoskeleton rearrangement of K7721 cells (Fig. 3C). To confirm the involvement of Src activation in CD147 regulating Rac1 activity, we measured the Rac1-GTP level in CD147-overexpressing cells. As shown in Fig. 3D, CD147 overexpression increased the level of Rac1-GTP, however, Src I-1 treatment abolished the promoting effect of CD147 overexpression on Rac1activity, indicating that CD147 promotes Rac/WAVE signaling through Src activation. Overall, these data demonstrated that CD147 regulates cell motility by promoting the activation of Src in HCC cells.

### CD147 promotes the phosphorylation of Src by FAK

**Fig.4:**
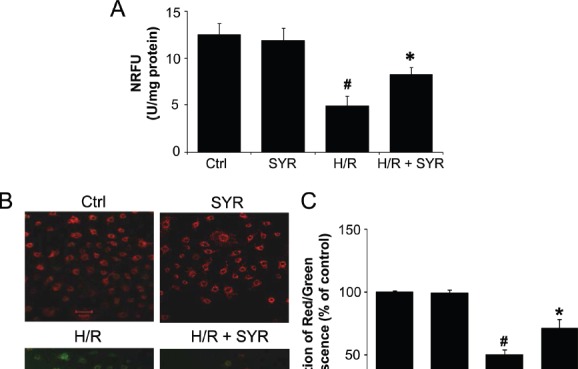
CD147 promotes Src activation through FAK (A) Src activity in CD147-pcDNA3.1-transfected 7721 cells was blocked with Src I-1. Left panel: representative image. Right panel: quantification. (B) Src activity in 7721 or CD147-pcDNA3.1-transfected 7721 cells was blocked with FAK inhibitor (PF573,228). Left panel: representative image. Right panel: quantification. (C) Src activity in CD147-pcDNA3.1-transfected 7721 cells was blocked with FAK inhibitor-14 (Y-15). Left panel: representative image. (D) Src activity in CD147-pcDNA3.1-transfected 7721 cells was blocked with FAK siRNA (si-FAK). Left panel: representative image. Right panel: quantification. The bars represent each sample performed in triplicate, and the error bars indicate ± SD. ** P < 0.01, * P < 0.05, # P > 0.05, by one-way ANOVA.

To investigate the correlation between CD147 and Src, we first evaluated Src activity in 7721 cells overexpressing CD147. pY416-Src was increased when CD147 was overexpressed in 7721 cells, and this effect was significantly decreased after treatment with Src I-1 (Fig. 4A). Our previous studies demonstrated that CD147 facilitates FAK phosphorylation at Y397 by interacting with α3β1 integrin [25]. Here, we found that Src activity could be inhibited with the FAK inhibitor and that the enhanced Src activity resulting from CD147 overexpression could be inhibited with either PF573,228 (Fig. 4B) or Y-15 (Fig. 4C). We recapitulated this effect using FAK siRNA (si-FAK) which was designed to knock-down FAK (Fig 4D). These results suggest that CD147 can promote the activation of Src through the integrin/FAK signaling pathway in HCC cells.

### DOCK8 is a Rac1 GEF involved in the mesenchymal movement regulated by CD147 signaling

**Fig.5:**
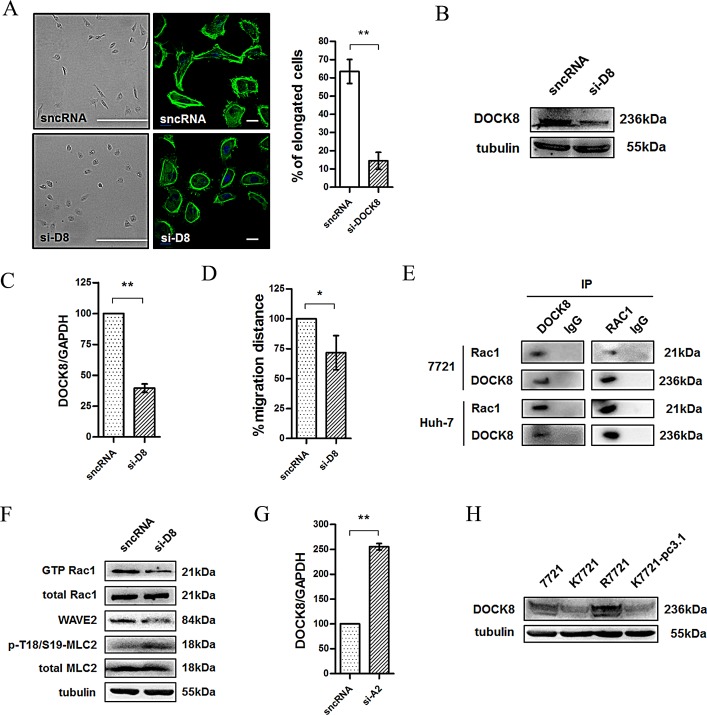
DOCK8 is a Rac1 GEF involved in the Src-regulated Rac/WAVE signaling pathway (A) Images (Scale bar = 500μm) and confocal microscopy images (Scale bar = 20μm) of the DOCK8 siRNA (si-D8)-induced morphological changes in 7721 cells. Green: actin; Blue: DAPI. Left panel: representative image. Right panel: quantification. (B) DOCK8 was detected using western blotting after the transfection of 7721 cells with DOCK8 siRNA. (C) The DOCK8 mRNA level was detected using real-time PCR after the transfection of 7721 cells with si-D8. (D) Effects of si-D8 on cell motility of 7721 cells. Cells were transfected with si-D8 prior to use. (E) Rac1 immunoprecipitated with DOCK8. (F) Rac1 activity, WAVE2 expression and MLC2 activity in 7721 and 7721-siD8 cells. (G) The mRNA level of DOCK8 was detected using real-time PCR after the transfection of 7721 cells with ANXA2 siRNA (si-A2). (H) DOCK8 expression was examined in total lysates of SMMC-7721, K7721, R7721 and K7721-pcDNA3.1 cells using western blotting. The bars represent each sample performed in triplicate, and the error bars indicate ± SD. ** P < 0.01, * P < 0.05, by t-test (A, C, D, G).

As neither CD147 nor Src is a GEF or GAP, Rac1 activity cannot be directly regulated by CD147 or Src. To identify the key molecules that directly regulate Rac1 activity in HCC cells, we screened the GEFs in 7721 cells by transfecting these cells with siRNAs targeting GEFs from the DOCK family. Strikingly, most of 7721 cells shifted from an elongated morphology to a rounded morphology (Fig. 5A) following the silencing of DOCK8 (Fig. 5B and 5C), which also led to attenuated cell motility (Fig. 5D). In order to investigate the interaction between Rac1 and DOCK8, we undertook co-immunoprecipitation assays. Protein expression of the two molecules in 7721 and Huh-7 cells was detected by western blotting. As shown in Figure 5E, DOCK8 or Rac1 was then found to co-immunoprecipitate with each other in 7721 and Huh-7 cell lysates, indicating that DOCK8 can physically interact with Rac1 and DOCK8 is very likely to be a GEF for Rac1 in HCC cells. To confirm this result, we evaluated Rac1 activity and the expression of WAVE2, which is located downstream of Rac1 in the mesenchymal movement signaling pathway. As shown in Fig. 5F, knockdown of DOCK8 resulted in a significant down-regulation of Rac1-GTP and WAVE2 expression, which implied that DOCK8 may regulate Rac1 signaling in HCC cells. Because Rho-ROCK signaling-mediated phosphorylation of MLC2 has been shown to be a key determinant of actomyosin contractility, we also investigated whether DOCK8 affected the Rho signaling pathway. Phospho-MLC2 (p-T18/S19-MLC2) was significantly increased after knockdown of DOCK8, which implied that DOCK8 promoted Rac1 signaling, but inhibited Rho signaling, in HCC cells. In addition, we previously reported that Annexin II is an activator of Rho signaling [16], we found that the mRNA level of DOCK8 was significantly up-regulated after Annexin II knockdown in 7721 cells (Fig. 5G). Furthermore, CD147 knockout cells showed substantially reduced DOCK8 expression compared to 7721 or R7721 cells (Fig. 5H). Together, these findings demonstrated that DOCK8 serves as a key Rac1 GEF involved in regulating Rac/Rho signaling, CD147-mediated cytoskeletal rearrangement, and cell movement in HCC cells.

### Src promotes STAT3 phosphorylation leading to enhanced DOCK8 transcription and expression

**Fig.6:**
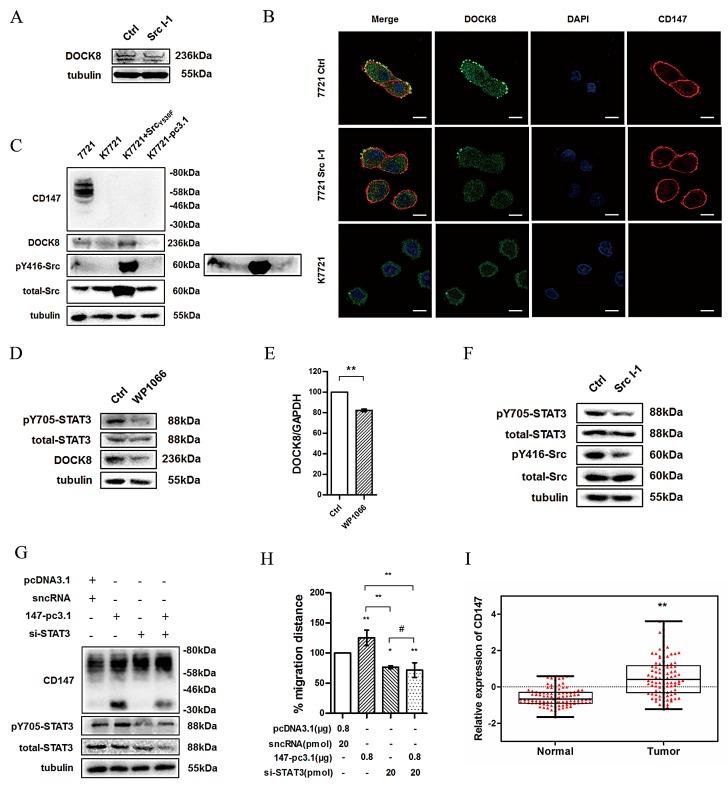
Src promotes DOCK8 expression via enhancing STAT3 phosphorylation (A) DOCK8 expression was detected using western blotting after treatment of 7721 cells with Src I-1. (B) Confocal microscopy images of 7721, 7721 treated with Src I-1 and K7721 cells. Red: CD147; Green: DOCK8; Blue: DAPI. Scale bar = 20μm. (C) DOCK8 expression and Src activity were examined in total lysates of 7721, K7721, K7721 transfected with Src_Y530F_ and K7721-pcDNA3.1 cells (K7721 cells were transfected with pcDNA3.1 vector as a mock control for R7721) using western blotting. Phosphorylation of Src at Tyr416 can also be seen in the overexposed panel (right).(D) Phosphorylation level of STAT3 and DOCK8 expression were detected using western blotting after treatment of 7721 cells with WP1066. (E) The DOCK8 mRNA level was detected using real-time PCR after treatment of 7721 cells with WP1066. (F) Phosphorylation level of STAT3 was detected using western blotting after treatment of 7721 cells with Src I-1. (G) Phosphorylation level of STAT3 and CD147 expression were determined in 7721 cells overexpressing CD147 and/or transfected with STAT3 siRNA. (H) The effects of CD147 overexpression and/or STAT3 silencing on cell motility of 7721 cells. (I) CD147 expression using normalized microarray gene expression data. The bars represent each sample performed in triplicate, and the error bars indicate ± SD. * P < 0.05, ** P < 0.01, # P > 0.05, by unpaired t-test (E), one-way ANOVA (H) and paired t-test (I).

Signal transducer and activator of transcription 3 (STAT3) integrates signals from cytokines and growth factors into transcriptional responses in target cells. It is an important regulator of cancer cell survival and inflammation [26, 27]. And activated STAT3 promotes the transcription of target genes by phosphorylating and translocation from cytoplasm into nucleus. Recent report revealed that CD147 acts as an upstream activator in STAT3-mediated pancreatic tumor development by forming a signaling complex with CD44s [28]. Katz et al. showed that the invasion of breast cancer is associated with up-regulation of STAT3 and Rac1 activity [29]. We therefore hypothesized that CD147 may promote DOCK8 transcription by enhancing STAT3 phosphorylation. First we explored whether DOCK8 expression is affected by Src activity. As expected, DOCK8 expression in cells treated with Src I-1 was also significantly reduced compared to that in control cells (Fig. 6A). Specific accumulation of DOCK8 occurred at the leading edge of moving cells, and this accumulation could be attenuated by Src I-1 or CD147 deficiency (Fig. 6B). These results were also observed in HepG2 and Huh-7 cell lines (data not shown). In addition, the decrease of DOCK8 induced by CD147 deletion could be reversed by expression of Src dominant-positive mutant (Src_Y530F_) (Fig. 6C). Then we treated cells with WP1066, a STAT3 pathway inhibitor, results showed that either the protein (Fig. 6D) or the mRNA level of DOCK8 (Fig. 6E) were decreased compared with control groups. Furthermore, the phosphorylation of STAT3 could be inhibited by Src I-1 (Fig. 6F). To determine whether depletion of STAT3 affects CD147-mediated migration, 7721 cells were co-transfected with CD147-pcDNA3.1 and STAT3 siRNA (Fig. 6G). As shown in Fig. 6H, STAT3 silencing blocked the CD147-mediated cell motility. Notably, phospho-STAT3 was significantly increased when CD147 was overexpressed. All of these data suggest that CD147 promotes Src-mediated STAT3 phosphorylation, which results in enhanced DOCK8 transcription and expression.

We have shown so far that CD147 promotes HCC cells movement. We investigated whether CD147 could be regulated during HCC development and progression. We analyzed CD147 mRNA expression utilizing published data from public database Gene Expression Omnibus (GEO). As shown in Fig 6I, increased CD147 expression was found in HCC tissues compared with normal liver tissues. These data suggest that there is an overall upregulation of CD147 expression levels and CD147 plays an important role during HCC progression.

## DISCUSSION

HCC is a malignant tumor with a high frequency of relapse and metastasis. Moreover, despite recent improvements in long-term survival rates, HCC patient prognosis remains poor [30]. One major hallmark of an aggressive solitary HCC is its ability to metastasize. Thus, understanding the mechanisms that underlie this process may promote the development of effective approaches to reduce HCC mortality.

Enhanced cell motility is a common feature of tumor metastasis. To migrate, cells undergo dynamic rearrangements of their actin cytoskeleton to form protrusive structures and generate the intracellular forces required for cell translocation. Actin-based cell motility relies on actin as well as numerous actin-interacting proteins and a wide variety of signaling molecules such as kinases and phosphatases that drive the dynamics of the actin system and govern its spatial organization [31].

The Src tyrosine kinase is widely recognized as a key mediator in cytoskeleton organization. Signals initiated by ECM-integrin interactions are transduced into cells through the activation of integrin-associated FAK and Src [32]. Moreover, Src has been shown to be involved in the regulation of Rho-dependent motility and remodeling of the actin cytoskeleton. Recent work from Sebastian et al. Further demonstrates that treatment with the Src inhibitor Dasatinib results in increased RhoA activity and diminished Rac activity in synovial sarcoma cells [33]. Khusial et al. reported that Src stabilizes the expression of Robo1 by activating the Abl kinase, and increases Rac1 activity to promote tumor cell migration [34]. The results of our present study show that RhoA activity is negatively correlated with the phosphorylation level of Src at Y416. On one hand, Src activity is positively correlated with Rac1 activity and WAVE2 expression, whereas on the other hand, Src plays a negative regulatory role in Rho/ROCK signaling. Enhanced Rac1 activity promotes mesenchymal-type cell movement and suppresses amoeboid cell movement by maintaining a low phosphorylation level of MLC2, which is consistent with results from previous studies [16].

Accumulating evidence indicates that DOCK family proteins are critical regulators of the small GTPase Rac during several fundamentally important biological processes, such as cell motility and phagocytosis. In mammalian cells, the DOCK family is encoded by at least 11 genes that can be further divided into 4 subgroups. Previous research have identified several novel homologues of DOCK180 and found that many of them directly bind to and exchange GDP for GTP both *in vitro* and *in vivo* on either Rac or another Rho-family member, Cdc42. [35]. To explore the mechanisms behind the positive regulation of Rac1 activity, we screened the DOCK180 family of GEFs and identified DOCK8 as a GEF for Rac1 that plays a key role in Src-induced activation of Rac during HCC metastasis. Since the initial report of DOCK8-deficient patients in 2009, DOCK8 has been shown to be essential for the survival of peripheral T cells and memory CD8^+^ T cells [36-38]. DOCK8 is present in lamellipodia and other areas that undergo dynamic actin reorganization [39], and it was also shown that the cellular role of DOCK8 in NK cell-mediated cytotoxicity is achieved, in part, through integrin-mediated adhesion to target cells and by polarization of F-actin and lytic granules at the NK cell cytotoxic synapse [40]. However, the role of DOCK8 in cancer cells remains undefined. Our current study revealed that Src is activated by the CD147-FAK signaling pathway and subsequently up-regulates the expression of DOCK8. It has also been reported that the activity of Rac1 is stimulated by tyrosine phosphorylation of p130Cas, which is an FAK-associated adaptor protein, and that a second adaptor, CRK, can be recruited to phosphorylate p130Cas [41]. Therefore, we hypothesize that the p130Cas-CRK complex brings the CRK-associated Rac GEF DOCK8 to sites of CD147-integrin signaling, and DOCK8-activated Rac1 can then stimulate actin polymerization and membrane protrusions, leading to cell motility and invasion. Interestingly, Rac1 inhibition leads to increased Src phosphorylation at Y416, but not at Y527. Conversely, Rac1 activation results in reduced phosphorylation of Src at Y416, but not at Y527 (Supplementary Fig. 1), indicating the existence of a feedback loop between Rac1 and Src. In fact, we previously demonstrated a positive feedback loop between Rac1 activation and CD147 expression [16]. These feedback loops may help explain the function of CD147 in cytoskeleton reorganization and play an important role in promoting HCC progression.

Previous studies have shown that CD147 interacts with the integrins α3β1 and α6β1 in HCC cells and activates the downstream FAK-PI3K-Ca^2+^ and FAK-paxillin pathways, thus contributing to the processes of cell adhesion, proliferation, differentiation, apoptosis, and tumor progression [42, 43]. Moreover, the interaction of CD147 with the integrin β1 subunit can be competitively blocked with the GRGDS peptide, which inhibits downstream FAK signal transduction and actin cytoskeleton rearrangement [25]. Many Rac GEFs have also been reported to be activated by PI3K/PIP3 signaling [44].

**Fig.7:**
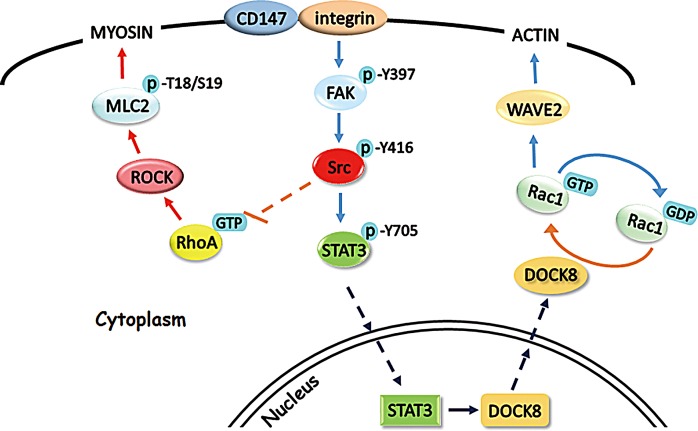
Schematic representation of the major molecular mechanisms of CD147 in regulating hepatocellular carcinoma cells motility FAK, focal adhesion kinase; DOCK8, dedicator of cytokinesis 8; ROCK, Rho-kinase; MLC2, myosin light chain 2; STAT3, signal transducer and activator of transcription 3.

As a protease-inducer, CD147 could stimulate the surrounding fibroblasts and endothelial cells to produce matrix metalloproteinases (MMPs) in autocrine and paracrine fashions [11, 15, 45, 46]. Recently, multiple studies have provided evidences that CD147 could regulate tumor angiogenesis by stimulating MMPs and VEGF production in tumor and stromal cells [47-50]. Consistent with our findings that CD147 functions in the interconversion between amoeboid and mesenchymal movements in HCC cells (Fig. 7), which is considered a dynamic process in the metastasis of tumor cells, previous work in our lab reported that CD147 promotes the epithelial-mesenchymal transition (EMT) during HCC progression [51]. This finding may provide another piece of evidence supporting the function of CD147 in cytoskeleton rearrangement and mesenchymal movement in HCC cells.

In summary, our studies identify CD147 as a novel regulator of Rac1 activity that acts through promoting STAT3 phosphorylation and DOCK8 expression, following modulation of Src activation by the integrin/FAK signaling pathway. Together with our previous results, these suggest that CD147 is an important regulator of the interconversion between amoeboid and mesenchymal movements and is involved in the invasion and metastatic processes of HCC cells. As a novel therapeutic target in the management of HCC, developing drugs that target CD147 may represent a promising strategy for HCC therapy.

## Materials and Methods

### Cell lines

Five human HCC cell lines were used: 7721 (SMMC-7721), HepG2, Huh-7, K7721, and R7721. SMMC-7721 and HepG2 cells were purchased from the Institute of Cell Biology, Academic Sinica (Shanghai, China). K7721 cells (7721 CD147^−/−^) and R7721 cells (CD147 is stably expressed in K7721 cells) were developed and preserved in our laboratory. Huh-7 cells were purchased from the Cell Bank of the JCRB (Tokyo, Japan).

### Materials

Antibodies specific for CD147 (sc-9754), α-tubulin (sc-8035), WAVE2 (sc-10392), donkey anti-goat IgG-FITC (sc-2024) and FAK Inhibitor 14 (Y-15) (sc-203950) were purchased from Santa Cruz (Dallas, Texas); phospho-FAK (Tyr397) (3283), phospho-Src (Tyr416) (2013), phospho-myosin light chain 2 (Thr18/Ser19) (3674), Src (2109), and myosin light chain 2 (3672) antibodies were purchased from Cell Signaling Technology (Boston, MA); DOCK8 (11622-1-AP) and STAT3 (60199-1-Ig) antibodies were obtained from Proteintech (Wuhan, China); phospho-STAT3 (Tyr705) (ab76135) antibody was obtained from Abcam (Cambridge, UK); phospho-Src (Tyr527) (orb14869) antibody was obtained from Biorbyt LLC (San Francisco, CA); and Alexa 594-conjugated goat anti-mouse IgG and Alexa Fluor 488 phalloidin were purchased from Invitrogen (Carlsbad, CA). The Src kinase inhibitor Src I-1, FAK inhibitor PF573,228 (Sigma, St. Louis, MO) and STAT3 inhibitor WP1066 (Merck Millipore, Darmstadt, GER) were also used. Src I-1 was used at 300 nM, PF573,228 at 300 nM, Y-15 at 10 μM and WP1066 at 5 μM. The Cell Proliferation Reagent WST-1 was purchased from Roche (Mannheim, GER).

### Cell Culture and Plasmids Transfection

Cells were cultured in RPMI 1640 medium supplemented with 10% FBS, 1% penicillin/streptomycin, and 2% L-glutamine at 37°C in a humidified atmosphere of 5% CO_2_. Huh-7 cells were cultured in DMEM medium supplemented with 10% FBS. Plasmids were transfected using Lipofectamine2000 reagent according to the manufacturer's instructions (Invitrogen, Carlsbad, CA).

Full-length CD147 was cloned into pcDNA3.1 (147-pc3.1) as previously prescribed [52]. Full-length pp60c-Src was cloned into pcDNA3.1 (Src-pc3.1) using primers as follows: forward primer, 5′-AGAATTCATGGGTAGCAACAAGAGCAA GCCCAAG-3′, and reverse primer, 5′-ATACTCGAGCTAGAGGTTCTCCCCGGGCT-3′. To generate Src_Y530F_ with a mutated tyrosine phosphorylation site, primers used were as follows: forward primer, 5′-CGGGCTGGAACTGGGGCTCGGTGG-3′, and reverse primer, 5′-CCACCGAGCCCCAGTTCCAGCCCG-3′.

### RNA interference

Chemically synthesized, double-stranded siRNAs were purchased from Shanghai GenePharma Co., Ltd (Shanghai, China). Sequence for CD147 siRNA (si-147) is 5′-GUUCUUCGUGAGUUCCUCtt-3′ [11]. siRNA for FAK (sc-35353) was purchased from Santa Cruz (Dallas, Texas), siRNAs for DOCK1 to DOCK11 were designed and synthesized by GenePharma Company. Lipofectamine2000 reagent was employed according to the manufacturer's instructions (Invitrogen, Carlsbad, CA). Silencer negative control siRNA (sncRNA) was used as a negative control.

### Western blotting

HCC cells were harvested in a lysis buffer, the protein quantification was determined by BCA Protein Assay Kit (Thermo Scientific, Waltham, MA) and equal amounts of cellular proteins were subjected to 8%-12% SDS-PAGE separation. Proteins were transferred to polyvinylidene fluoride (PVDF) microporous membranes (Millipore, Boston, MA). Western blotting was performed with the DAB Horseradish Peroxidase Color Development Kit (Beyotime, Shanghai, China) with horseradish peroxidase-conjugated secondary antibodies (Sigma, St. Louis, MO). Tubulin was chosen as an internal control and the blots were probed with mouse α-tubulin mAb.

### RhoA and Rac1 activity assay

RhoA and Rac1 activity measurements were carried out according to the Rho (BK036) and Rac (BK035) activation assay kit instructions (Cytoskeleton, Denver, CO).

### Immunofluorescence

Twenty-four hours after siRNA transfection or inhibitor treatment, HCC cells were harvested and allowed to attach for 24 h to Matrigel-pre-coated cell culture dishes with glass bottoms (NEST Biotechnology, Wuxi, China). After washing twice with PBS, the cells were fixed in paraformaldehyde in PBS, permeabilized with 0.1% Triton X-100, and blocked with 1% BSA in PBS for 1 h. The dishes were first incubated with the indicated antibodies for 1 h, washed twice with PBS, and then incubated with Alexa 488-phalloidin solution (1:40) and the corresponding FITC-conjugated secondary antibodies for 30 min in the dark. Cell nuclei were dyed with DAPI (Vector Laboratories, Burlingame, CA). After washing, the dishes were covered with an anti-fade reagent to prevent quenching of the fluorophores, and the cells were visualized using an A1R-A1 confocal laser microscope system (Nikon, Tokyo, Japan).

### Real-time RT-PCR analysis

Total RNA was extracted using an E.Z.N.A.® Total RNA Kit II (Omega Bio-Tek, Norcross, GA) and reversely transcribed into cDNA using a ReverTra Ace-a Kit (Toyobo, Osaka, Japan). SYBR-Green real-time RT-PCR was performed using the Stratagene Mx3005P sequence detection system (Agilent Technologies, Santa Clara, CA) and SYBR Premix EX Taq II (2×) (Takara, Shiga, Japan). GAPDH mRNA was used to normalize the RNA inputs. All primers were synthesized by Shanghai Sangon Biological Engineering Technology & Services Co., Ltd.

### *In vitro* cell invasion and migration assays

These assays were performed using chambers containing polycarbonate filters (8 μm pore size; Merck Millipore, Darmstadt, GER). The upper side of a polycarbonate filter was either coated (invasion) or not coated (migration) with Matrigel (BD Bioscience, San Jose, CA) to form a continuous, thin layer. HCC cells (1×10^5^) were re-suspended in 300 μL of 0.1% serum medium with or without Src I-1 and then added to the upper chamber. The lower chamber was filled with 10% FBS medium (200 μL). After a 24-h (invasion) or 8-h (migration) incubation and removal of the cells from the upper chamber of the filter with a cotton swab, the cells on the underside were stained and counted.

### *In vitro* wound healing assay

Twenty-four hours after siRNA transfection or inhibitor treatment, the cells were harvested and seeded in 12-well plates until confluent. Scratching of the monolayer with the tip of a pipette was performed to wound the monolayer. The cells were then washed with serum-free medium and cultured in RPMI 1640 medium with 0.1 % FBS. Photomicrographs at 10× objective magnification were obtained at various time points (0 h and 24 h), and the relative migration distance was calculated using the following formula: the relative migration distance (%) = 100 (AX–BX) / (A blank–B blank), where A is the width of the cell wound before incubation, and B is the width of the cell wound after incubation. The results are expressed as the mean ± SD.

### Proliferation assay

HCC cells (1×10^3^) were seeded in 96-well plates and incubated in 100 μL serum-free medium 1640. Cells were then treated with or without Src I-1 for 0h, 24h and 48h. Then add 10 μL/well WST-1 and incubate for 120 minutes at 37°C. Plates were then read by a scanning multiwell spectrophotometer by measuring the absorbance of the dye with a wavelength of 450 nm and a reference wavelength of 690 nm. Same volume of culture medium and WST-1 was used as blank control. Three different experiments were performed for each experimental condition.

### Co-immunoprecipitation assay

The interaction of DOCK8 with Rac1 in SMMC-7721 and Huh-7 cells was detected using a ProFound Mammalian Co-Immunoprecipitation Kit (Pierce Biotechnology, IL, USA), according to the manufacturer's instructions. Briefly, cells (1×10^7^) were lysed using IP Lysis/Wash Buffer. The lysate was collected onto a coupling resin that was pre-bound with 20 μg of the mouse anti-human DOCK8 mAb, rabbit anti-human Rac1 mAb or IgG isotype control, followed by four washes with IP Lysis/Wash Buffer. The coupling resin was then washed with elution buffer, and aliquots of the eluent were analysed by western blotting.

### Analysis of CD147 expression from human databases

Gene expression data of human HCC samples from published microarray study (GEO Accession number GSE22058) was used to analyze the expression of CD147 in HCC progression.

### Statistical analysis

All experiments were performed in triplicate, and the results were expressed as the mean ± SD. Statistical significance was determined using the GraphPad Prism V5.0 software (GraphPad Software, La Jolla, CA). Differences were deemed significant if P < 0.05. One-way ANOVA was performed for multiple comparisons; two-tailed *t* test was performed for other experiments to compare the mean values. ** indicates P < 0.01, * indicates P < 0.05, and # indicates P > 0.05. Error bars indicate ± SD.

## SUPPLEMENTARY MATERIAL AND FIGURE


