# Supplementary material for: CD147 promotes Src-dependent activation of Rac1 signaling through STAT3/DOCK8 during the motility of hepatocellular carcinoma cells
Source: Oncotarget. 2014 Nov 16;6(1):243–57. doi: 10.18632/oncotarget.2801 (PMC4381592; doi:10.18632/oncotarget.2801)
Supplement: Supplementary file 1 [file oncotarget-06-243-s001.pdf]

CD147 promotes Src-dependent activation of Rac1 signaling through STAT3/DOCK8 during the motility of hepatocellular carcinoma cells

Supplementary Material

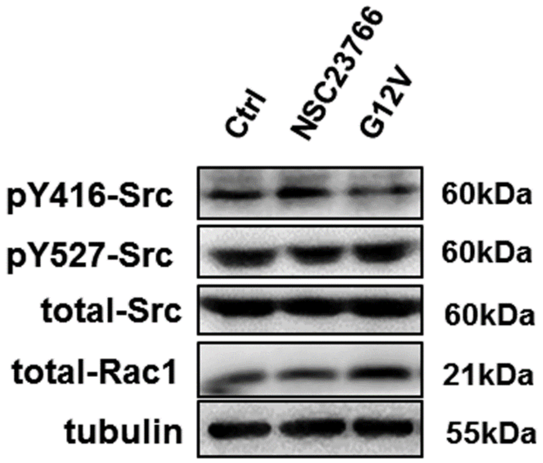

Supplementary Fig. 1: Phosphorylation of Src was assessed using western blotting in 7721 cells treated with Src inhibitor (NSC23766) or transfected with G12V mutant.
